# Forecasting the final disease size: comparing calibrations of Bertalanffy–Pütter models

**DOI:** 10.1017/S0950268820003039

**Published:** 2020-12-28

**Authors:** Norbert Brunner, Manfred Kühleitner

**Affiliations:** Department of Integrative Biology and Biodiversity Research (DIBB), University of Natural Resources and Life Sciences (BOKU), A-1180 Vienna, Austria

**Keywords:** Bertalanffy–Pütter model (BP-model), epidemic trajectory, final disease size, forecast, weighted least-squares

## Abstract

Using monthly data from the Ebola-outbreak 2013–2016 in West Africa, we compared two calibrations for data fitting, least-squares (*SSE*) and weighted least-squares (*SWSE*) with weights reciprocal to the number of new infections. To compare (in hindsight) forecasts for the final disease size (the actual value was observed at month 28 of the outbreak) we fitted Bertalanffy–Pütter growth models to truncated initial data (first 11, 12, …, 28 months). The growth curves identified the epidemic peak at month 10 and the relative errors of the forecasts (asymptotic limits) were below 10%, if 16 or more month were used; for *SWSE* the relative errors were smaller than for *SSE*. However, the calibrations differed insofar as for *SWSE* there were good fitting models that forecasted reasonable upper and lower bounds, while *SSE* was biased, as the forecasts of good fitting models systematically underestimated the final disease size. Furthermore, for *SSE* the normal distribution hypothesis of the fit residuals was refuted, while the similar hypothesis for *SWSE* was not refuted. We therefore recommend considering *SWSE* for epidemic forecasts.

## Introduction

Epidemiology uses a wide variety of mathematical tools to model the spread of infectious diseases. ‘Mathematical models […] take many forms, depending on the level of biological knowledge of processes involved and data available. Such models also have many different purposes, influencing the level of detailed required’ [1]. Examples of the most common model types that use highly aggregated data (infection counts over time) are trend models, as considered in this paper (another example is GGM of [2]), and compartmental dynamical systems models (including the classical continuous and deterministic SIR in [3], or stochastic SIR and SEIR in [4] or [5], respectively). By contrast, individual-based simulation models describe the interactions of large numbers of individuals and therefore need detailed information to characterise them [6]. This paper focuses on trend-models with sigmoidal (S-shaped) growth curves, as these have been useful for practitioners. For example, the simple logistic growth model correctly predicted the slowing down of the 2013–2016 Ebola outbreak in West Africa [7–11].

In this paper, we considered the more general Bertalanffy–Pütter (BP) differential equation (details in the section ‘Method’) for modelling epidemic trajectories. For an illustration, we fitted BP models to data from the 2013–2016 Ebola outbreak in West Africa, using the method of least-squares (nonlinear regression). In order to assess the practical significance of this model-fitting exercise, we used initial data from the Ebola outbreak to forecast the final disease size. Forecasting the final size of an epidemic is an important task for modelling; underestimating it may lead to a false sense of security.

In previous papers, we have studied the BP equation for modelling the growth of tumours, chicken, dinosaurs and fish [12–15] and we found that it was a versatile tool that resulted in significant improvements in the fit of the model to the data, when compared to previous results in the literature. However, in that papers we also found that the least-squares method underestimated the potential of further growth. We therefore proposed a calibration that for the considered size-at-age data provided more realistic asymptotic size estimates.

For the Ebola data, we observed the same problem; using ordinary least-squares the final disease size was underestimated. As to the reason, ordinary least-squares assumes equal variances while animal sizes and epidemic data displayed size-dependent variances (heteroscedasticity). However, the pattern of heteroscedasticity was different: although for the size of smaller/larger animals the variance was lower/higher, for epidemic data (total disease counts) the variance was highest at the epidemic peak (most new cases) and low at the initial and final phases. For the Ebola data, we therefore used a weighted least-squares method with a new weight function (details in the section ‘Method’): Using this calibration, prediction of the final disease size became reliable for the late phase of the outbreak (after it has peaked).

## Method

### Materials

The data and the results of the optimisations were recorded in spreadsheets (Microsoft Excel); see the Supporting information. The computations used Mathematica 12.0 software (Wolfram Research). We used commercially available PCs and laptops (Intel i7 core). For them, CPU-time for fitting the BP model to monthly data was 1 week per dataset (less for *SSE*). However, for weekly data and *SWSE*, data fitting used 8 weeks. Therefore, the paper reports the results for the monthly data.

### Data

**Fig. 1.**
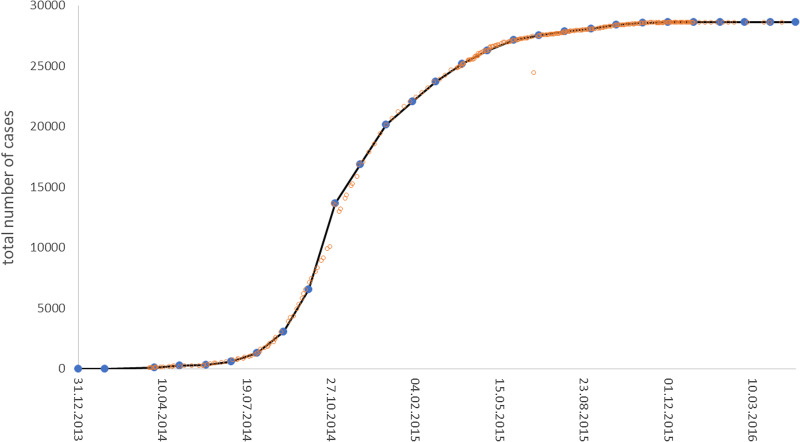
Total weekly and monthly count of Ebola cases in West Africa; blue dots connected with a black line are the values of Table 1 and brown rings are the counts from CDC [16]; plotted using MS Excel.

CDC [16] compiled data from three countries of West Africa, based on weekly Ebola reports by the WHO (Fig. 1) that counted the confirmed, probable and suspected infections and fatalities in each country. CDC [16] added the numbers of infections since the start of the outbreak to the number of total cases.

Unfortunately, there were some flaws in the data. First, there were false-positive and false-negative diagnoses: initially Ebola was diagnosed as fatal diarrhoea (December 2013 and January 2014) and at the peak of the outbreak villages reported Ebola cases that were later falsified, but the total counts in the reports were not corrected retrospectively. Second, the published data contained typos, incorrect arithmetic and an obvious outlier at 24 June of 2015. (We suppose that it resulted from a typo: 24 472 instead of 27 472.) We therefore aggregated the CDC list to Table 1, which eliminated data uncertainty as much as possible without altering the data. It also reduced random fluctuations. Nevertheless, it still was representative of the raw data (Fig. 1).

**Table 1.** Total monthly count of Ebola cases in West Africa

Month	Cases^a^	Month	Cases^a^	Month	Cases^a^	Month	Cases^a^
0^b^	1	8	3052	15	25 178	22	28 547
1	5	9	6553	16	26 298	23	28 601
3	120	10	13 676	17	27 145	24	28 604
4	242	11	16 899	18	27 540	25	28 603
5	309	12	20 171	19	27 840	26	28 603
6	599	13	22 057	20	28 065	27	28 610
7	1322	14	23 694	21	28 388	28	28 616

a For each month, the table records the maximum of the total number of cases reported from Guinea, Liberia and Sierra Leone since the beginning of the outbreak.

b Month 0 is December 2013 (index patient).

*Source*: Adapted from [16].

In addition, we also fitted the BP models to the full set of data from CDC [16] about West Africa. As amongst the 265 data-points (brown rings in Fig. 1) there were contradictory values for 19 April, 3 May and 30 June of 2015, we took the larger counts (reducing the size of the dataset to 262 data-points) and left the data otherwise unaltered (not removing the outlier).

### The BP model class

The BP differential equation (1) describes the cumulative count of infected individuals, *y*(*t*), as a function of time, *t*, since the start of an infection. It uses five model parameters that are determined from fitting the model to cumulative epidemiological data; non-negative exponent pair *a* < *b*, non-negative scaling constants *p* and *q* and the initial value *y*(0) = *c* > 0:1
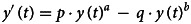
Equation (1) appeared originally in Pütter [17]; von Bertalanffy [18, 19] popularised it as a model for animal growth. In epidemiology, and for *a* ≤ 1, this model has been proposed as the generalised Richards model [20, 2^1^]. It can be solved analytically, although in general not by means of elementary functions [22].

**Fig. 2.**
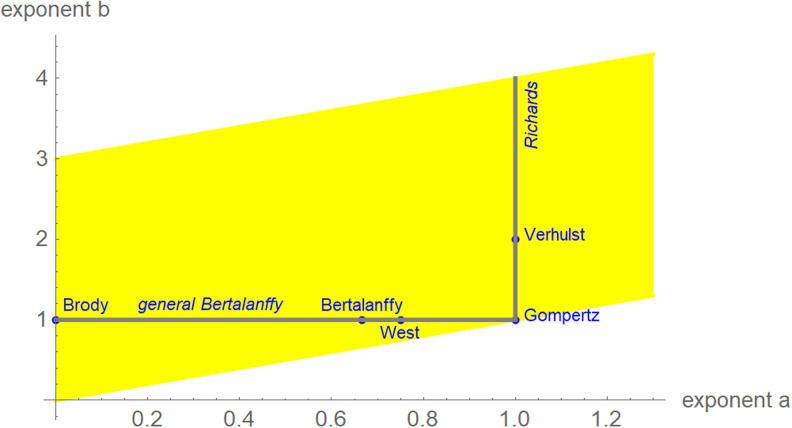
Named models (blue) and initial search region (yellow) of BP models (plot using Mathematica 12.0): 0 ≤ *a* ≤ 1.3, *a* < *b* ≤ *a* + 3, step size in both directions 0.01. When needed, the grid was extended.

We interpret equation (1) as a model class, where each exponent pair (*a*, *b*) defines a unique model *BP*(*a*, *b*) in the BP class of models; *BP*(*a*, *b*) has three free parameters (*c*, *p*, *q*). Equation (1) then includes several trend models that have been used to describe epidemic trajectories [23–25], such as the Brody [26] model of bounded exponential growth *BP*(0, 1), [27] logistic growth *BP*(1, 2), the model *BP*(2/3, 1) of von Bertalanffy [18] or the model *BP*(3/4, 1) of West *et al*. [28]. Also, the Gompertz [29] model fits into this scheme: it is the limit case *BP*(1, 1), with a different differential equation, where *b* converges to *a* = 1 from above [30]. Equation (1) also includes several classes of models, such as the generalised Bertalanffy model (*b* = 1 with *a* variable) and the Richards [31] model (*a* = 1 with *b* variable). However (Fig. 2), when compared to the range of models searched for this paper (yellow area), these named models appear as rather exceptional.

Some authors added further assumptions about the parameter values in order to simplify the model (e.g. setting *c* = 1 in advance for the indicator case). We do not consider such simplifications.

### Model calibration

The (ordinary) least-squares method is common in epidemiology for large case counts, as for the present data [32]. It measures the goodness of the fit to the data by means of *SSE*, the sum of squared errors (fit residuals). In the present context, we sought parameters *a*, *b*, *c*, *p* and *q* so that for the solution *y*(*t*) of equation (1) the following sum *SSE* was minimised, whereby (*t_i_*, *y_i_*) were the first *n* total case counts *y_i_* at time *t_i_* (here 10 ≤ *n* ≤ 28):2
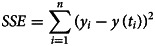
An implicit assumption of the least-squares method is a normal distribution of the errors: in formal terms, *SSE* is equivalent to the maximum-likelihood estimate under the assumption of normally distributed data with expected value *y*(*t*) and time-independent variance *s* > 0. If this assumption is not satisfied, as for the animal size-at-age data or the outbreak data, other methods of calibration need to be considered. For the animal data we could work well under the assumption of a log-normal distribution, where the variance increases with the size. However, for the Ebola data (total case counts) this assumption was problematic, too, as the variance was maximal at an intermediate stage (epidemic peak).

We therefore developed another measure for the goodness of fit, weighted least-squares that aimed at finding parameters to minimise the following sum of weighted squared errors *SWSE*:3
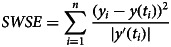
This weight function tolerates a higher variability in the total count during the epidemic peak. In formal terms, *SWSE* identifies the maximum-likelihood estimate under the assumptions that the data are normally distributed with expected value *y*(*t*) and a variance *s*⋅*y*′(*t*) with *s* a constant. (Thus, the variance of the total counts is assumed to be proportional to the absolute value of the derivative of *y*. This derivative corresponds to the number of new cases.)

In the following subsections, we will explain our notation using *SSE*; the same definitions will also apply to *SWSE*.

### Optimisation

For model class (1) standard optimisation tools (e.g. Mathematica: NonLinearModelFit) may not identify the best-fit parameters (numerical instability). To overcome this difficulty, we used a custom-made variant of the method of simulated annealing [33] which solves the optimisation problem to a prescribed accuracy [12]. Note that using simulated annealing to optimise all five parameters of equation (1) at once may not always come close to the optimum parameters, because the region of nearly optimal parameters may have a peculiar pancake-like shape ([15]; extremely small in one direction and extremely large in other ones).

We confined optimisation to a grid (Fig. 2). For each exponent pair on the grid 0 ≤ *a* ≤ 1.3 and *a* < *b* ≤ 3 with distance 0.01 in both directions we searched for the best-fit model *BP*(*a*, *b*); i.e. we minimised *SSE* for the model *BP*(*a*, *b*). Thus, we defined the following function *SSE_opt_* on the grid:4
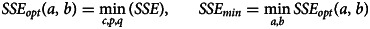
Summarising this notation, for each exponent pair (*a*, *b*) on the grid we identified the best-fit model *BP*(*a*, *b*) with certain parameters *c*, *p*, *q* and the corresponding least sum of squared errors *SSE_opt_*(*a*, *b*). These values were recorded in a spreadsheet. Finally, the best-fit model had the overall least sum of squared errors (*SSE_min_*). It minimised the function *SSE_opt_* at the optimal exponent pair (*a_min_*, *b_min_*). From the above-mentioned spreadsheet we could then read off the other parameters *p_min_*, *q_min_*, *c_min_* that minimised *SSE_opt_*(*a_min_*, *b_min_*) of the model *BP*(*a_min_*, *b_min_*). However, if that optimal exponent pair was on the edge of the search grid, then we extended the search grid and continued the optimisation. (Otherwise, if the optimal exponent pair was surrounded by suboptimal ones, then we stopped the search for an optimum.)

### Model comparison

For each exponent pair (*a*, *b*) of the search grid we identified a best-fitting model *BP*(*a*, *b*), whose fit was given by *SSE_opt_*(*a*, *b*) of formula (4). Amongst these models we selected the model with the least *SSE_opt_*. In the literature, there are various alternative criteria for the comparison of models, amongst them the root mean squared error *RMSE* and the Akaike's [34] information criterion (*AIC*). *RMSE* is the square-root of *SSE*/*n* and *AIC* = *n* ⋅ ln (*SSE*/*n*) + 2*K*, where *n* is the number of data-points (between 10 and 28 for the monthly truncated data), *K* = 4 is the number of optimised parameters of the model *BP*(*a*, *b*) with a given exponent pair (counting *c*, *p*, *q* and *SSE*), and *SSE* = *SSE_opt_*(*a*, *b*). As an additional measure to compare the goodness of fit, we used formula (5) for the relative Akaike weight:5
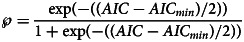


This formula has been interpreted as probability, 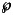, that the (worse) model with higher *AIC* would be ‘true’, when compared to the model with the least *AIC* [35, 36]. Thereby (citation from [37], on p. 272, referring to information theory of [38]) ‘true’ means that the model ‘is, in fact, the K-L best model for the data… This latter inference about model selection uncertainty is conditional on both the data and the full set of a priori models [in formula 5: the models with *AIC* and with *AIC_min_*] considered’. Furthermore, as does *AIC*, the Akaike weight assumes a normal distribution of the (weighted) fit residuals.

Using *SSE* or *AIC* is only meaningful with reference to a fixed dataset (different models are fitted to the same data), whereas *RMSE* and 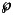 make also sense, if we consider datasets with different sizes, as for the forecasts, where we successively use truncated initial data with 10, 11, …, 28 months. Thereby, we prefer the probabilities for their more intuitive appearance. Note that 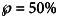 is the maximal value that can be attained by 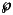 (comparison of the best-fit model with its copy).

### Multi-model approach

**Fig. 3.**
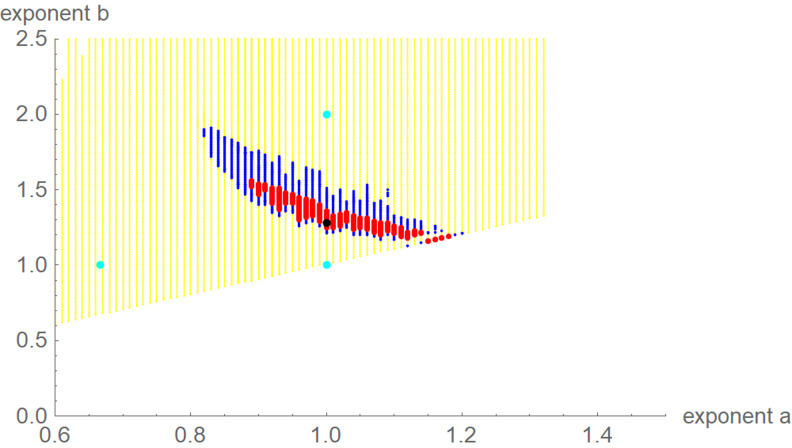
Optimal exponent-pair (black) for fitting the data of Table 1 with respect to *SSE*; red and black dots for 245 and 1330 exponent pairs with up to 17% and 50% higher *SSE*; exponent-pairs of the Bertalanffy, Gompertz and Verhulst models (cyan) and part of the search grid (yellow).

For each exponent pair (*a*, *b*) of the search grid we identified the best-fit growth curve *y_a,b_*(*t*) for the model *BP*(*a*, *b*); its parameters *p*, *q* and *c* were optimised according to formula (4). Thereby, we could observe a high variability in the best-fit exponents: even for exponent pairs (*a*, *b*) with notable differences from the optimal exponent pair (*a_min_*, *b_min_*) the best-fit growth curve *y_a,b_*(*t*) barely differed from the data. To describe this phenomenon, we used the following terminology: a model *BP*(*a*, *b*) and its exponent pair (*a*, *b*) are *u*-near-optimal with model-uncertainty *u*, if *SSE*_*opt*_(*a*, *b*) ≤ (1 + *u*) ⋅ *SSE*_*min*_. Figure 3 illustrates this concept by plotting near-optimal exponent pairs for different levels *u* = 0.17 and *u* = 0.5 of model-uncertainty (red and blue dots).

Using formula (5), we translated the level *u* of near optimality into a probability 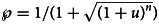. For example, if we wish to consider models (exponent pairs) with a probability of at least 10%, this corresponds to a model uncertainty of at most *u* = 0.55 for the 10-month data (*n* = 10) and *u* = 0.17 for the 28-month data.

For each dataset, we then may ask about the forecasts that would be supported by models with e.g. 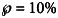 or higher, discarding other possible forecasts as unlikely, given the data. Thus, given a model probability, 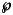, we studied the forecasts that came from the ensemble of the growth curves *y_a,b_*(*t*) corresponding to the near-optimal models *BP*(*a*, *b*) with that probability or higher. For example, for each of these *y_a,b_*(*t*) we estimated its final disease size by the asymptotic limit. The upper and lower bounds for these estimates defined a *prediction interval* that informed about the likely disease size. Note that this is an analysis of model uncertainty, not of data uncertainty, whence the prediction interval is *not* a confidence interval. (Confidence intervals assess data uncertainty by means of simulations that add random errors to the data and use best-fit functions to compute bounds for the confidence interval. For prediction intervals, different models are fitted to the same data.)

## Results

### Best fits

Table 2 identified the optimal parameters of BP models that were fitted by ordinary least-squares, equation (2) for *SSE*, to initial segments of the monthly data, meaning the data of Table 1 for the first 10 ≤ *n* ≤ 28 months. As slight changes in the parameter values could result in highly suboptimal growth curves, from which standard optimisation tools could not find back to the region of near optimality, certain parameters were given with an accuracy of 30 or more decimals. Table 3 identified the optimal parameters for initial segments, using the method of weighted least-squares, formula (3) for *SWSE*.

**Table 2.** Parameters for the best-fit (*SSE*) model (1) to the data up to the indicated month

Month^a^	Model parameters^b^					Goodness of fit^c^		Asymptotic limit^c^
	*a_min_*	*b_min_*	*c_min_*	*p_min_*	*q_min_*	*SSE_min_*/10^6^	RMSE	
10	1.19	1.42	17.7741	0.317228	0.0228423	0.0153	39.09	*93 647*
11	1.02	6.6	8.45046	0.663536	1.657 × 10^−24^	0.0211	43.84	16 976
12	1.38	1.6	27.263	0.123849	0.0138776	1.4188	343.85	21 006
13	1.29	1.35	4.84703	0.819036	0.448672	1.9064	382.94	22 711
14	1.2	1.29	1.33673	1.12273	0.452778	2.4962	422.26	24 105
15	1.02	1.4	0.0781697	1.44265	0.0306548	3.5057	483.44	25 220
16	1.15	1.16	0.188038	12.6072	11.3871	4.5167	531.31	26 333
17	1.1	1.25	0.35805	1.40607	0.304888	5.7890	583.55	26 655
18	1.0	1.32	0.0562717	1.68091	0.0640025	6.3774	595.23	27 256
19	1.03	1.3	0.184543	1.46154	0.0925867	6.9879	606.45	27 416
20	1.02	1.29	0.159357	1.54147	0.0972962	7.3897	607.85	27 788
21	0.95	1.38	0.0186636	1.96722	0.0241207	7.9605	615.69	27 878
22	1.0	1.31	0.123787	1.60994	0.0672919	8.3679	616.73	28 047
23	1.07	1.23	0.461196	1.49859	0.290988	8.7655	617.34	28 106
24	1.03	1.26	0.23758	1.54609	0.146399	8.8929	608.72	28 240
25	1.02	1.27	0.217945	1.56039	0.120352	9.0691	602.30	28 256
26	0.99	1.3	0.101559	1.70456	0.0710008	9.1356	592.76	28 362
27	1.03	1.25	0.229343	1.58429	0.166104	9.2035	583.84	28 318
28	1.0	1.28	0.135994	1.67323	0.0947475	9.2829	575.79	28 414

a This indicates the data from month 0 to the displayed month.

b The table reports the parameters based on the best-fit grid-point exponent pairs (the overall optima were slightly different).

c Estimates above the actual count of 28 616 cases in italics.

**Table 3.** Best-fit parameters with respect to *SWSE* for the data up to the indicated month

Month	*a_min_*	*b_min_*	*c_min_*	*p_min_*	*q_min_*	*SWSE_min_*	*RMSE*	Asymptotic limit
10	1.05	9.6	14.022600	0.515153	1.08640 × 10^−36^	133.934	3.65971	14 881
11	1.05	6.12	14.005700	0.515379	1.83792 × 10^−22^	133.997	3.49021	16 996
12	1.16	2.04	20.008300	0.277716	0.0000429172	520.806	6.58790	21 412
13	1.23	1.65	19.336400	0.226581	0.0033374200	668.755	7.17235	22 986
14	1.21	1.52	13.764400	0.310600	0.0135567000	1055.060	8.68110	24 392
15	1.13	1.45	6.163090	0.551204	0.0213620000	1785.130	10.9091	25 791
16	1.02	1.53	2.362370	0.945249	0.0052132000	2605.260	12.7604	26 811
17	1.04	1.35	1.190240	1.102900	0.0463179000	3139.260	13.5890	27 622
18	1.00	1.39	0.763762	1.278370	0.0235874000	3425.320	13.7948	27 931
19	1.06	1.32	2.046030	1.018100	0.0709812000	3537.840	13.6456	28 096
20	0.99	1.46	1.549080	1.178370	0.0095493700	4010.670	14.1610	28 157
21	0.96	1.36	0.202642	1.697710	0.0280686000	4329.330	14.3582	28 451
22	1.00	1.25	0.196457	1.723510	0.1325430000	4595.860	14.4535	28 591
23	1.02	1.22	0.292313	1.724230	0.2213960000	4733.780	14.3463	*28 650*
24	0.94	1.43	0.456417	1.668570	0.0109219000	5501.110	15.1398	*28 657*
25	0.93	1.51	1.223280	1.567290	0.0040738800	6211.630	15.7628	*28 651*
26	0.90	1.59	0.771413	1.825950	0.0015353400	6535.000	15.8539	*28 637*
27	0.93	1.43	0.254940	1.798650	0.0106292000	5423.320	14.1726	*28 635*
28	0.90	1.61	1.144070	1.774490	0.0012154400	6846.480	15.6370	*28 630*

*Notes* as in Table 2 (referring to *SWSE* rather than to *SSE*).

**Fig. 4.**
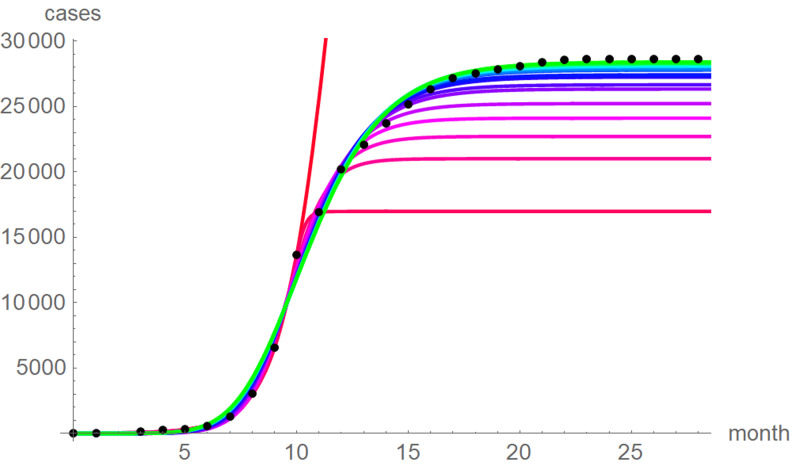
Monthly data (black dots) with the best-fit growth curves (*SSE*) for the data until month 10 (red), 11, … 28 (green); month 0 is December 2013. The best-fit parameters are from Table 2 (plotted using Mathematica 12.0).

**Fig. 5.**
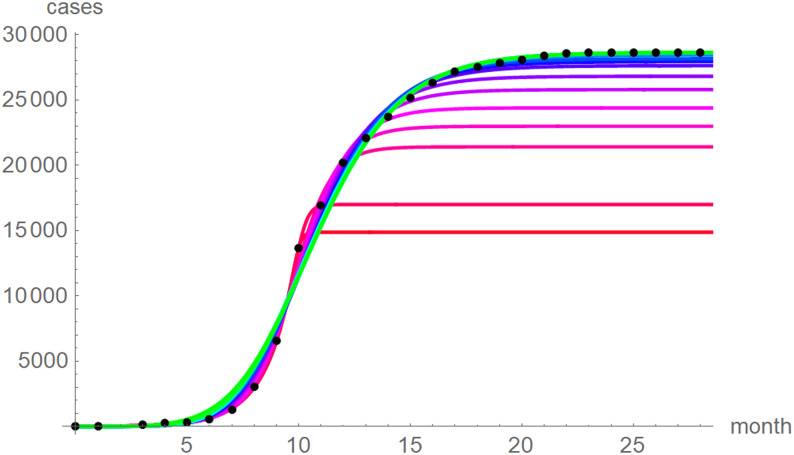
Monthly data (black dots) with the best-fit growth curves (*SWSE*) for the data until month 10 (red), 11, … 28 (green); month 0 is December 2013. The best-fit parameters are from Table 5 (plotted using Mathematica 12.0).

At a first glance, the growth curves for the same data looked similar, regardless of the method of calibration. Figure 4 plots the monthly data and the growth curves that were fitted to the initial segments by means of ordinary least-squares and Figure 5 plots the monthly data and the best-fit curves using weighted least-squares. The two figures look alike and for both methods good forecasts (asymptotic limits in Tables 2 and 3) for the final disease size of 28 616 cases could be obtained from the data truncated at month *n*, *n* between 16 and 28, whereby the forecasts using weighted least-squares were more accurate: the relative error of the forecasts was 1–8% for *SSE* and 0–6% for *SWSE*. All growth curves were bounded, whereby for *SSE* the first curve (data truncated at month 10) exceeded the data and the following curves approached the data from below; all asymptotic limits remained below the actual final disease size. For *SWSE* the growth curves initially approached the data from below and starting with month 23 their asymptotic limits were slightly above the final disease size.

**Fig. 6.**
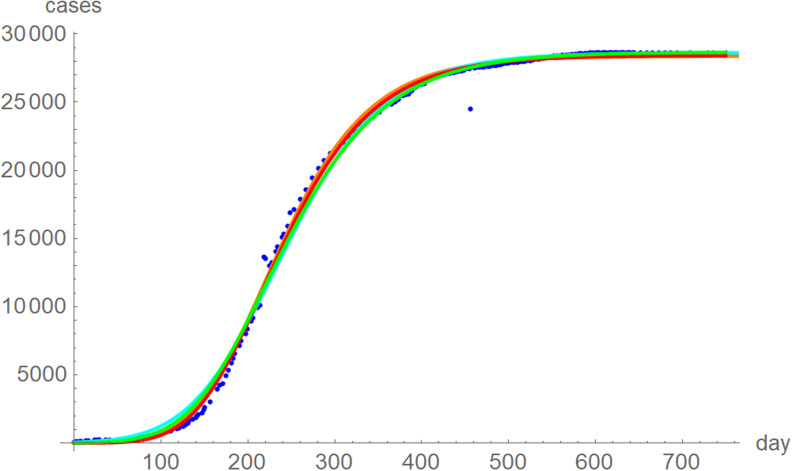
Weekly data (blue dots, with correction of three inconsistencies), best-fitting growth curve to these data using *SSE* (red) and *SWSE* (green) and best-fitting growth curves fitted to the monthly data using *SSE* (orange) and *SWSE* (cyan), whereby at day *x* we evaluated the growth function at month (*x* + 84)/30.4, because the daily data started later. The parameters are given in the Supporting information; plotted using Mathematica 12.0.

The best fit curves to the full data were analysed in more detail, including for the weekly data (details given in the Supporting information). Figure 6 plots the growth curves that were fitted to the full sets of monthly and weekly data, respectively, using the two calibrations. Again, there were only slight differences between the curves that used ordinary and weighted least-squares; for *SSE* and *SWSE* the asymptotic limits were below and above the final disease size, respectively. Furthermore, the growth curves for the weekly and monthly data that were calibrated by the same method were largely overlapping.

**Fig. 7.**
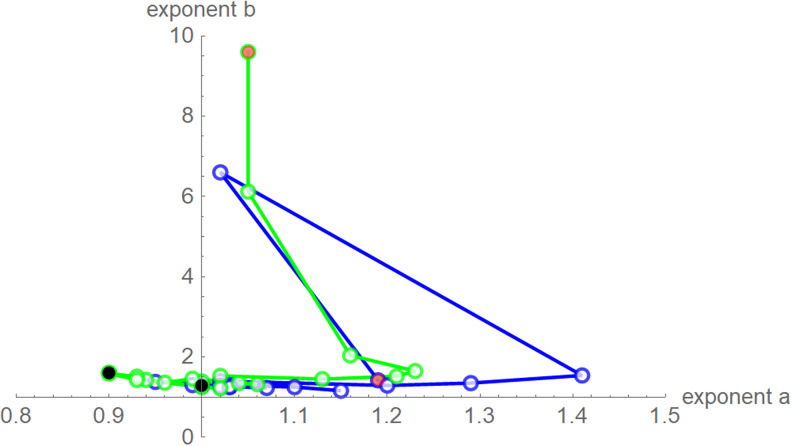
Best-fit exponent pairs for the truncated monthly data using *SSE* (blue) from Table 2 and *SWSE* (green) from Table 3. Lines connect exponent pairs for successive data, starting with the exponent pair for the 10-month data (red) and ending with the exponent pair for the 28-month data (black); plotted using Mathematica 12.0.

Despite these similarities there were obvious differences for the exponent pairs that were computed using different calibrations; Figure 7 plots them. Thereby, except for the *SSE* fit to the 28-month-data (Richards' model), all exponent pairs were clearly distinct from the exponent pairs of the named models mentioned in the section ‘Method’.

### Testing distribution assumptions

We now explain, why despite the apparent utility of the ordinary least-squares method we will recommend our method of weighted least-squares. One reason was that for the present data its implicit distribution assumption was not outright false: the criteria weights for *SWSE* were motivated from the observation that the variance of the cumulative count depends on the number of new cases (derivative of the cumulative count), as it was low at the beginning and end of an outbreak and maximal at the peak of the epidemics, while *SSE* assumed equal variances for all data. We checked these distribution assumptions for the full dataset by testing for *SSE* the normal distribution hypothesis for the residuals and for *SWSE* the normal distribution hypothesis for the residuals divided by the square root of *y*′.

Table 4 summarises the results. We tested the fits to the full set of monthly data and for both calibration methods we took the residuals from the best-fit models that we obtained from additional simulated annealing steps (parameters given in the Supporting information). We applied a set of the most common distribution fit tests to the residuals; they relate to different aspects for normality [39]. For ordinary least-squares (*SSE*) with one exception (skewness) the tests supported the conclusion that at 95% confidence the residuals were not normally distributed (*P*-values below 5%). Hence, an implicit assumption of this method (and of the AIC) was refuted. For weighted least-squares (*SWSE*) this difficulty did not occur: none of the tests rejected the assumption of a normal distribution for the residuals divided by the square root of the best-fit trajectory *y*′, as all tests had sufficiently large *P*-values (20–70%).

**Table 4.** Testing the normal distribution hypothesis for *SSE* and *SWSE*

Test	*SSE*		*SWSE*	
	Statistic	*P*-value (%)	Statistic	*P*-value (%)
Anderson and Darling	0.843113	2.9	0.395597	37.5
Baringhaus and Henze	0.616057	4.4	0.203591	42.3
Cramér and von Mises	0.138078	3.4	0.0561172	43.3
Jarque and Bera	10.6558	3.6	2.03096	24.4
Kolmogorov and Smirnov	0.183602	1.7	0.129329	26.9
Kuiper	0.250117	2.8	0.178515	31.9
Mardia combined	10.6558	3.6	2.03096	24.4
Mardia kurtosis	2.07855	3.8	0.370169	71.1
Mardia skewness	1.38398	23.9	1.16285	28.1
Pearson *χ*^2^	12.0	3.5	6.28571	27.9
Shapiro and Wilk	0.911888	2.2	0.961703	38.2
Watson *U*^2^	0.137992	2.4	0.0524089	45.8

*Note*: Based on the best-fit growth curves *y* to the 28-month data, distribution-fit tests were applied to the residuals (*SSE*) and to the residuals divided by the square-root of *y*′ (*SWSE*), respectively. The best-fit parameters are described in the text.

### Multi-model comparisons

In this section, we outline another drawback of the ordinary least-squares method (*SSE*): using *SSE*, for the present data good conservative forecasts for the estimated final disease size could not be obtained with models that fitted well to the data. Thus, paradoxically, a prudent forecaster using ordinary least-squares would deliberately use models that are highly unlikely in terms of their probability 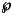 (Akaike weight) of equation (5). By contrast, for weighted least-squares (*SWSE*) the prediction intervals for the asymptotic limit contained the final disease size, whereby the prediction intervals used only likely models (high probability 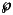).

Tables 5 and 6 inform about the 10% prediction intervals for the asymptotic limits, using truncated monthly data, best-fit models using ordinary and weighted least-squares, respectively, and assuming for all models a probability 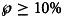. If the data were truncated at month 10, then for both methods of calibration the 10% prediction intervals were useless: for *SSE*, already the lower estimate was too pessimistic and for *SWSE* the interval was unbounded above. For *SSE* there were only two data with 10% prediction intervals that contained the final disease size of 28 616 cases: the data truncated at months 26 and 27. By contrast, using *SWSE* for all datasets truncated at any of the months 21–28, the 10% prediction intervals contained the final disease size.

**Table 5.** 10% prediction intervals for asymptotic limits (*SSE*) and probabilities for predicting the month-28 count

Month^a^	Asymptotic limit^b^		Probability^c^ (%)	Month^a^	Asymptotic limit^b^		Probability^c^ (%)
	Low	High			Low	High	
10	*29 806*	*2 706 403*	8	20	27 165	28 217	0.75
11	15 721	∞	25.11	21	27 377	28 429	1.01
12	19 768	23 488	0.13	22	27 613	28 480	1.4
13	21 571	24 705	0.03	23	27 799	28 594	3.8
14	23 038	25 398	0.01	24	27 874	28 539	3.65
15	24 143	26 359	0.03	25	27 940	28 557	9.19
16	25 191	27 366	0.17	26	28 005	28 621	8
17	25 953	27 597	0.38	27	28 098	28 630	25.11
18	26 423	27 903	0.55	28	28 145	28 587	0.13
19	26 893	28 139	0.65				

a Truncated data from month 0 to the displayed month.

b Minimum and maximum of the asymptotic limits for models with 10% probability to be true, based on *SSE*, with limits above 28 616 cases displayed in italics.

c Minimum of the maximal probabilities of models *BP*(*a*, *b*), whose trajectories at month 28 were above or below the actual count of 28 616 cases.

**Table 6.** 10% prediction intervals for asymptotic limits (*SWSE*) and probabilities for predicting the month-28 count

Month^a^	Asymptotic limit^b^		Probability^c^ (%)	Month^a^	Asymptotic limit^b^		Probability^c^ (%)
	Low	High			Low	high	
10	*12 899*	∞	50	20	28 034	28 426	0.42
11	16 918	*17 752*	0.003	21	28 304	*28 646*	14.72
12	20 529	23 309	0.11	22	28 468	*28 691*	37.52
13	22 341	23 996	0.01	23	28 527	*28 731*	48.25
14	23 959	25 209	0.01	24	28 584	*28 716*	41.04
15	25 259	26 672	0.1	25	28 596	*28 694*	46.34
16	26 364	27 584	0.33	26	28 591	*28 679*	47.42
17	27 183	28 396	2.8	27	28 594	*28 673*	45.35
18	27 581	28 426	1.76	28	28 600	*28 664*	46.62
19	27 882	28 464	3.79				

*Notes* as for Table 5 (referring to *SWSE* rather than to *SSE*).

We applied the notion of the prediction interval also to other predictors, such as the forecasted disease size at month 28 (and in the Supporting information, Tables S1–S3: best-fit model parameters for *SSE* and *SWSE* and coordinates of the inflection point). Tables 5 and 6 inform about these intervals in an abbreviated form, informing about the least probability 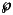 so that the actual disease size of 28 616 cases could be found in the 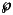 prediction interval. For example, the month-10 entry of Table 5 was obtained as follows: the best-fit model was *BP*(1.07, 1.29) and for its epidemic trajectory *y*_1.07, 1.29_(*t*) the value *y*(28) was above the actual disease size, while for the model *BP*(1.19, 1.42) and for its best fitting epidemic trajectory *y*_1.19, 1.42_(*t*) the value *y*(28) was below the actual disease size. The probabilities for these models were 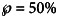 and 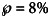 (this was the maximal probability for such a model); the minimum of these probabilities was reported in the table. For ordinary least-squares, models with a probability below 10% were needed to enclose the actual disease size in the prediction interval. Specifically, for the full data (month 28), only unlikely trajectories (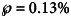, meaning 0.0013) exceeded the true count at month 28. Again, for weighted least-squares and all datasets truncated at any of the month 21–28, the 10% prediction intervals contained the final disease size. Furthermore, for the data truncated at any of the months 21–28, all likely growth curves (probability 10% to be true) deviated from the final case count by at most ±2%. Thereby, there were both likely curves above and below the final case count. Thus, other than for ordinary least-squares, for weighted least-squares there was no bias towards too optimistic forecasts, and forecasts were not too pessimistic, either.

## Discussion

Our results have several implications for practical applications. With respect to the choice of BP models, Viboud *et al*. [21] recommended to use only exponents *a* ≤ 1 to model the initial phases of epidemics. However, for most truncated data better fits were achieved for *a* > 1. Therefore, such a restriction appears premature.

Concerning the two methods (*SSE* and *SWSE*) for the calibration of the models, we observed that the best-fit BP models obtained by means of ordinary least-squares in general had asymptotic limits that were too low, when compared to the final disease size. For the modelling of epidemics this resulted in overly optimistic forecast of the final disease sizes. Better estimates for the final disease size could be obtained by weighted least-squares. Thereby, different types of data needed different weight functions. For epidemic data (total case counts) we recommend using as weight function the reciprocal of the derivative of the growth function; formula (3). This recommendation was supported by an analysis of the (weighted) fit residuals. The normal distribution assumption of the ordinary least-squares method was refuted, but the similar assumption for weighted least-squares was not refuted.

For the present data we concluded that a reasonable prognosis of upper and lower bounds of the final disease size was possible much earlier when using this weight function rather than ordinary least-squares, namely already at month 21 or later, while for ordinary least-squares it was not sure, if that prognosis was feasible at all. However (Supporting information, Table S3), as follows from the prediction intervals for the inflection point, the peak of the disease was attained around *t_infl_* ≈ 10 months regardless of the method of calibration. Thus, even with a suitable weight function the prognosis of the final disease size may be possible only at a surprisingly late phase of a disease.

Obviously, no calibration can extract more information about the future trajectories than is available from the given data. Forecasts using data from an early stage of epidemics prior to its peak (e.g. 10-month data) remained uninformative also with the new calibration. Thus, for precise forecasts about the final size enough data-points beyond the peak of the epidemics were needed to inform about how fast the epidemics slowed down. However, for the present data the proposed weight function had the advantage that its estimates of the final size of the disease were reliable earlier than for the ordinary least-squares method that currently is the standard method for such purposes.

## Conclusion

This paper proposes a new calibration of phenomenological models in the context of modelling infectious disease outbreaks. We recommend weighted least-squares using the reciprocals of the derivatives of the growth function (this corresponds to the numbers of new cases) as weights.

We tested this calibration for the modelling of the West African Ebola outbreak of 2013–2016 by means of the BP model. Although for these data the estimates remained comparable to those of the least-squares method, we conducted several tests (distribution of the fit residuals and forecasting intervals for estimating the final disease size) that confirmed that the new calibration was superior to ordinary least-squares.

## Data Availability

The comparisons of models are based on the data in Table 1. As explained in the section ‘Data’, these data are based on the raw data published in CDC [16]; see Figure 1 for a comparison of Table 1 with the raw data from the literature. In addition, the computations of the paper generated new data about the best-fit parameters; for details see the Supporting information.

## Supplementary material

For supplementary material accompanying this paper visit https://doi.org/10.1017/S0950268820003039. click here to view supplementary material
