# Supplementary material for: Forecasting the final disease size: comparing calibrations of Bertalanffy–Pütter models
Source: Epidemiol Infect. 2020 Dec 28;149:e6. doi: 10.1017/S0950268820003039 (PMC8057487; doi:10.1017/S0950268820003039)
Supplement: Supplementary file 1 [file S0950268820003039sup.zip › S0950268820003039sup002.docx]

**Supporting Information** to the paper
Forecasting the final disease size: Comparing calibrations of Bertalannfy-Pütter models

By: Norbert Brunner and Manfred Kühleitner

**BP-models (2.3).** The expected final size of an epidemic is estimated by the asymptotic limit as ${y_{max}= (\frac{p}{q})}^{\frac{1}{b-a}}$, as for this value of *y* the right-hand side of (1) vanishes. The peak of an epidemics (maximum of *y´*) is attained at the inflection point (Hsieh et al., 2010), whose *y*-value for BP-models is $y_{infl}=\left( \frac{a}{b} \right)^{\frac{1}{b-a}}\cdot y_{max}$; from this the *t*-value *t_infl_* is obtained by numerical equation solving. Further, for *a* = 1, the parameter *p* is known as the “intrinsic growth rate”. Hsieh et al. (2004) used it to estimate the basic reproduction number as *R*_0_ = *e^p^*^⋅^*^T^*, *T* the generation time for disease transmission; e.g. *T* = 12 days = 0.4 month for Ebola (Chowell & Nishiura, 2014).

**Optimization (2.5).** For each dataset we optimized between 8950 (18-month data) to 71,663 (16-month data) grid-point models *BP*(*a*, *b*) for *SSE* and between 4077 (24-month data) and 66,079 (10-month data) models for *SWSE*, as this optimization was more time consuming. Some authors used empirical interpretations of the model to obtain approximate solutions to the parameter optimization. For example (approximating *SSE*), they applied a linear regression to fit, for each exponent-pair, a function $p\cdot y^{a}-q\cdot y^{b}$ to the numerical derivative *y´*(*t*) of the data (Lega & Brown, 2016; originally Walford, 1946 and Ricklefs, 1967). However, these approximations may be far from the best-fit parameters.

**Model Comparison (2.6)**. As we reduced optimization to the comparison of a finite set of given models (defined by a grid), for *AIC* we did not penalize the best-fit model *BP*(*a_min_*, *b_min_*) by counting the exponents as optimized parameters. (As seen below, the best-fit grid-exponents could indeed be further optimized.) Therefore, *BP*(*a_min_*, *b_min_*) had also the least *AIC_min_*. Insofar, in the present context all three measures for the goodness of fit provide the same information.

**Best fits (3.1).** The best fit curves to the full data were analyzed in more detail. Using ordinary least-squares, the model with the best fit to the 28-month-data with a grid-point exponent-pair was *BP*(1, 1.28) with the parameters in Table 2. Using 200,000 annealing steps, we improved this to *a* = 1.0036, *b* =1.28096, *c* = 0.134867, *p* = 1.65321, *q* = 0.0962188 and *SSE* = 9.2324⋅10^6^. Similarly, using weighted least-squares, the model with the best fit to the 28-month-data from grid optimization was *BP*(0.9, 1.61) with the parameters from Table 3, which we improved in 400,000 annealing steps to *a* = 0.909094, *b* = 1.62325, *c* = 1.35957, *p* = 1.65322, *q* = 0.00108528, with *SWSE* = 6845.5.

We repeated these computations for 262 weekly data about West Africa with the three corrections mentioned in Section 2.2. Using least-squares, the best-fit model (using grid-point-exponents) was *BP*(0.9, 1.33) with parameters *c* = 0.98, *p* = 0.097, *q* = 0.001 and *SSE* = 3.778⋅10^7^ (asymptotic limit: 28,420 cases). Using weighted least-squares, the best-fit model (grid-point-exponents) was *BP*(1.1, 1.11) with parameters *c* = 15.89, *p* = 0.4866, *q* = 0.4391 and *SWSE* = 948,446 (asymptotic limit: 28,676 cases). For the weekly data, the best-fit ordinary least-squares model of Liu et al. (2015) was in Richards’ class (*a* = 1); our *SSE*-model was not (*a* = 0.9). One reason for this difference was the outlier, which we did not remove, while Liu et al. (2015) had removed it. Further, the exponent-pairs for the weekly data differed from those for the monthly data. This was due to the different data (later start of the weekly data) and the different time scales.

For both methods of calibration (*SSE* and *SWSE*) the path described by the exponent-pairs corresponding to the truncated monthly data started erratic and later it appeared to approach the best-fit exponent-pairs for the full data. Thereby, for the exponent *a* the differences between the two methods of calibration were small (medians 1.03 and 1.02 and *p*-values 0.14 for a pairwise comparison by the sign test, 0.18 for the Mann-Whitney test for a different location and 0.64 for the Conover test of variance), while the exponents *b* differed significantly for the different methods of calibration (medians 1.3 and 1.46 and *p*-values 0.02, 0.002 and 0.04, respectively).

**References for the Supporting Information**

Chowell, G., Nishiura, H., 2014. Transmission dynamics and control of Ebola virus disease (EVD): a review. *BMC Medicine***12**, 196-212.

Hsieh, Y.H., Lee, J.Y., Chang, H.L., 2004. SARS epidemiology, logistic-type model, and cumulative case number. *Emerging Infectious Diseases* **10**, 1165-1167.

Hsieh, Y.H., Fisman, D.N., Wu, J.H., 2010. On epidemic modeling in real time: an application to the 2009 Novel A(H1N1) influenza outbreak in Canada. *BMC Research Notes* **3**, article 283.

Liu, W., Tang, S., Xiao, Y., 2015. Model Selection and Evaluation Based on Emerging Infectious Disease Data Sets including A/H1N1 and Ebola. *Computational and Mathematical Methods in Medicine*, published online: DOI 10.1155/2015/207105.

Lega, J., Brown, H.E., 2016. Data-driven outbreak forecasting with a simple nonlinear growth model. *Epidemics* **17**, 19-26.

Ricklefs, R.E., 1967. A graphical method of fitting equations to growth curves. *Ecology* **48**, 978-983.

Walford, L., 1946. A new graphic method of describing the growth of animals. *Biol. Bulletin* **90**, 141-147.

Table S1. 10%-prediction-intervals for model parameters, assuming *SSE*.

| Month^1^ | *a_min_* | | *b_min_* | | *c_min_* | | *p_min_* | | *q_min_* | |
| --- | --- | --- | --- | --- | --- | --- | --- | --- | --- | --- |
|  | low | high | low | high | low^2^ | high | low | high | low^2^ | high |
| 10 | 1.07 | 1.29 | 1.13 | 2.32 | 6.119 | 35.464 | 0.143 | 4.499 | 0^+^ | 4.499 |
| 11 | 0.96 | 1.09 | 4.6 | 11.99 | 2.077 | 21.64 | 0.377 | 1.088 | 0^+^ | 1.088 |
| 12 | 0.86 | 1.6 | 1.26 | 3.35 | 0^+^ | 121.737 | 0.016 | 6.174 | 0^+^ | 6.174 |
| 13 | 0.86 | 1.46 | 1.25 | 2.77 | 0^+^ | 71.253 | 0.051 | 6.346 | 0^+^ | 6.346 |
| 14 | 0.87 | 1.33 | 1.23 | 2.33 | 0^+^ | 35.878 | 0.133 | 7.317 | 0^+^ | 7.317 |
| 15 | 0.87 | 1.33 | 1.22 | 2.1 | 0^+^ | 24.127 | 0.235 | 7.842 | 0^+^ | 7.842 |
| 16 | 0.88 | 1.31 | 1.13 | 1.9 | 0.001 | 13.48 | 0.454 | 15.143 | 0^+^ | 15.143 |
| 17 | 0.87 | 1.22 | 1.19 | 1.87 | 0^+^ | 11.936 | 0.433 | 8.631 | 0^+^ | 8.631 |
| 18 | 0.87 | 1.21 | 1.18 | 1.77 | 0.001 | 4.489 | 0.646 | 9.008 | 0^+^ | 9.008 |
| 19 | 0.87 | 1.19 | 1.17 | 1.76 | 0.001 | 5.481 | 0.633 | 9.667 | 0^+^ | 9.667 |
| 20 | 0.86 | 1.21 | 1.16 | 1.72 | 0^+^ | 3.449 | 0.744 | 10.421 | 0^+^ | 10.421 |
| 21 | 0.87 | 1.2 | 1.17 | 1.7 | 0^+^ | 3.82 | 0.772 | 9.497 | 0.001 | 9.497 |
| 22 | 0.87 | 1.19 | 1.17 | 1.67 | 0.001 | 3.494 | 0.811 | 9.213 | 0.001 | 9.213 |
| 23 | 0.87 | 1.19 | 1.17 | 1.66 | 0.001 | 3.845 | 0.873 | 9.134 | 0.001 | 9.134 |
| 24 | 0.88 | 1.18 | 1.15 | 1.62 | 0.003 | 2.353 | 0.913 | 10.904 | 0.001 | 10.904 |
| 25 | 0.89 | 1.17 | 1.16 | 1.59 | 0.004 | 1.902 | 0.969 | 10.326 | 0.002 | 10.326 |
| 26 | 0.89 | 1.18 | 1.15 | 1.58 | 0.007 | 2.76 | 0.984 | 10.703 | 0.002 | 10.703 |
| 27 | 0.89 | 1.17 | 1.17 | 1.57 | 0.006 | 2.073 | 1.033 | 9.19 | 0.002 | 9.19 |
| 28 | 0.89 | 1.18 | 1.16 | 1.56 | 0.007 | 2.679 | 1.07 | 10.286 | 0.002 | 10.286 |

Notes: ^1^data from month 0 to the displayed month; ^2^0^+^ means a positive number, whose decimals are not displayed due to rounding.

Table S2. 10%-prediction-intervals for model parameters, assuming *SWSE*.

| Month^1^ | *a_min_* | | *b_min_* | | *c_min_* | | *p_min_* | | *q_min_* | |
| --- | --- | --- | --- | --- | --- | --- | --- | --- | --- | --- |
|  | low | high | Low | high | low^2^ | high | low | high | low^2^ | high |
| 10 | 0.98 | 1.26 | 0.99 | 12.14 | 5.036 | 30.786 | 0.169 | 0.898 | 0 | 0.898 |
| 11 | 0.99 | 1.15 | 3.04 | 7.1 | 6.022 | 28.939 | 0.251 | 0.821 | 0^+^ | 0.821 |
| 12 | 0.96 | 1.39 | 1.49 | 3.5 | 2.71 | 49.751 | 0.072 | 1.076 | 0^+^ | 1.076 |
| 13 | 0.97 | 1.31 | 1.51 | 2.65 | 2.45 | 41.781 | 0.11 | 1.051 | 0^+^ | 1.051 |
| 14 | 0.95 | 1.28 | 1.43 | 2.24 | 1.067 | 33.98 | 0.151 | 1.286 | 0^+^ | 1.286 |
| 15 | 0.89 | 1.27 | 1.38 | 2.09 | 0.075 | 30.356 | 0.2 | 2.009 | 0^+^ | 2.009 |
| 16 | 0.85 | 1.25 | 1.4 | 2.06 | 0.003 | 28.124 | 0.248 | 2.611 | 0^+^ | 2.611 |
| 17 | 0.85 | 1.19 | 1.27 | 1.89 | 0.001 | 20.628 | 0.366 | 2.709 | 0^+^ | 2.709 |
| 18 | 0.85 | 1.14 | 1.34 | 1.83 | 0.001 | 15.934 | 0.439 | 2.772 | 0^+^ | 2.772 |
| 19 | 0.85 | 1.13 | 1.29 | 1.77 | 0.001 | 12.937 | 0.51 | 2.757 | 0^+^ | 2.757 |
| 20 | 0.84 | 1.05 | 1.42 | 1.82 | 0.001 | 10.002 | 0.676 | 2.912 | 0^+^ | 2.912 |
| 21 | 0.85 | 1.09 | 1.33 | 1.67 | 0^+^ | 8.144 | 0.68 | 2.844 | 0.001 | 2.844 |
| 22 | 0.86 | 1.15 | 1.16 | 1.59 | 0.002 | 3.291 | 1.106 | 9.603 | 0.001 | 9.603 |
| 23 | 0.87 | 1.15 | 1.14 | 1.54 | 0.004 | 2.09 | 1.15 | 11.552 | 0.003 | 11.552 |
| 24 | 0.84 | 0.98 | 1.4 | 1.7 | 0.008 | 3.392 | 1.161 | 2.784 | 0^+^ | 2.784 |
| 25 | 0.83 | 0.96 | 1.47 | 1.8 | 0.026 | 4.773 | 1.161 | 2.828 | 0^+^ | 2.828 |
| 26 | 0.84 | 0.97 | 1.53 | 1.82 | 0.063 | 6.999 | 1.067 | 2.664 | 0^+^ | 2.664 |
| 27 | 0.84 | 1 | 1.36 | 1.66 | 0.002 | 4.419 | 1.024 | 2.903 | 0.001 | 2.903 |
| 28 | 0.83 | 1.07 | 1.37 | 1.85 | 0.087 | 6.315 | 0.756 | 2.699 | 0^+^ | 2.699 |

Notes as for Table S1 (referring to *SWSE* rather than to *SSE*).

Table S3. 10%-prediction-intervals for the inflection points.

| Month^1^ | *SSE* | | | | *SWSE* | | | |
| --- | --- | --- | --- | --- | --- | --- | --- | --- |
|  | *t_infl_* | | *y_infl_* | | *t_infl_* | | *y_infl_* | |
|  | low | high | low | high | low | high | Low | high |
| 12 | 9.4 | 9.8 | 9754 | 11,760 | 9.6 | 10.0 | 10,734 | 12,474 |
| 13 | 9.5 | 9.9 | 10,156 | 11,924 | 9.7 | 10.1 | 11,149 | 12,449 |
| 14 | 9.6 | 9.9 | 10,453 | 11,925 | 9.8 | 10.1 | 11,370 | 12,491 |
| 15 | 9.7 | 10.0 | 10,882 | 12,008 | 9.9 | 10.2 | 11,515 | 12,698 |
| 16 | 9.7 | 10.0 | 11,034 | 12,051 | 10.0 | 10.4 | 11,896 | 12,929 |
| 17 | 9.8 | 10.1 | 11,305 | 12,220 | 9.9 | 10.4 | 11,553 | 12,846 |
| 18 | 9.8 | 10.1 | 11,446 | 12,277 | 9.9 | 10.4 | 11,826 | 12,833 |
| 19 | 9.9 | 10.1 | 11,550 | 12,334 | 9.9 | 10.4 | 11,595 | 12,757 |
| 20 | 9.9 | 10.1 | 11,602 | 12,332 | 10.0 | 10.4 | 12,105 | 12,871 |
| 21 | 9.9 | 10.1 | 11,599 | 12,353 | 9.9 | 10.4 | 11,679 | 12,595 |
| 22 | 9.9 | 10.1 | 11,655 | 12,347 | 9.9 | 10.3 | 11,653 | 12,406 |
| 23 | 9.8 | 10.1 | 11,629 | 12,365 | 9.9 | 10.2 | 11,631 | 12,256 |
| 24 | 9.9 | 10.1 | 11,667 | 12,318 | 10.0 | 10.4 | 12,043 | 12,627 |
| 25 | 9.9 | 10.1 | 11,702 | 12,293 | 10.1 | 10.5 | 12,287 | 12,909 |
| 26 | 9.9 | 10.1 | 11,651 | 12,298 | 10.1 | 10.5 | 12,421 | 13,001 |
| 27 | 9.9 | 10.1 | 11,659 | 12,264 | 10.0 | 10.3 | 11,847 | 12,547 |
| 28 | 9.9 | 10.1 | 11,698 | 12,271 | 10.1 | 10.6 | 12,380 | 13,062 |

Note: ^1^ data from month 0 to the displayed month, whereby for month 10 and 11 there were models without inflection points.
